# Pulsed field ablation of a focal atrial tachycardia from the superior vena cava in proximity to the phrenic nerve: a case report

**DOI:** 10.1093/ehjcr/ytaf048

**Published:** 2025-01-31

**Authors:** Konstantinos Tampakis, Evangelia-Erasmia Papakonstantinou, Alexandros Sykiotis, Sokratis Pastromas, George Andrikopoulos

**Affiliations:** Department of Electrophysiology and Pacing, Henry Dunant Hospital Center, 107 Mesogeion ave, Athens 11526, Greece; Department of Electrophysiology and Pacing, Henry Dunant Hospital Center, 107 Mesogeion ave, Athens 11526, Greece; Department of Electrophysiology and Pacing, Henry Dunant Hospital Center, 107 Mesogeion ave, Athens 11526, Greece; Department of Electrophysiology and Pacing, Henry Dunant Hospital Center, 107 Mesogeion ave, Athens 11526, Greece; Department of Electrophysiology and Pacing, Henry Dunant Hospital Center, 107 Mesogeion ave, Athens 11526, Greece

**Keywords:** Atrial tachycardia, Superior vena cava, Phrenic nerve injury, Radiofrequency ablation, Pulsed-field ablation

## Abstract

**Background:**

Right phrenic nerve (PN) injury is a major complication of thermal ablation of atrial tachycardias (ATs) originated from the superior vena cava (SVC).

**Case summary:**

We report the case of a 41-year-old female patient admitted for catheter ablation of a frequent paroxysmal AT resistant to antiarrhythmic drugs. Electroanatomical activation map demonstrated a focal origin located at the lateral aspect of the SVC, ∼17 mm above the breakthrough of the sinus node wavefront. Importantly, high-output pacing from this site resulted in PN capture. To avoid PN injury, low-output radiofrequency (RF) ablation, with a power output limited to 20 W, was performed. However, this approach was insufficient to terminate AT. High-power RF applications in proximity to the PN were avoided and pulsed-field ablation (PFA) with a pentaspline catheter was chosen. The catheter was advanced into the SVC to the level of the earliest activation under fluoroscopic guidance and visualization within the mapping system. Two pairs of applications, in basket configuration, were delivered inside the SVC, rendering AT non-inducible while sinus node function was not compromised.

**Discussion:**

Phrenic nerve is vulnerable to injury during ablation within the SVC using thermal ablation modalities. Low-output RF ablation may be safe but less efficient. In contrast, non-thermal approaches such as PFA may be preferable to avoid damage to the collateral tissues as PN. Electroanatomical mapping may be important to avoid lesions in proximity to the sinus node.

**Learning points**

- Superior vena cava (SVC) has been recognized as a focal source of atrial ectopy initiating atrial tachycardia. Right phrenic nerve (PN) injury is one of the major complications of thermal ablation within the SVC.
- Low-output radiofrequency delivery may be safe but less efficient while non-thermal approaches such as pulsed-field ablation (PFA) may be preferable to avoid PN injury.
- More data needed for potential sinus node dysfunction after SVC PFA isolation while the role of electroanatomical mapping may be important to avoid lesions in proximity to sinus node.

## Introduction

Superior vena cava (SVC) has been recognized as a focal source of atrial ectopy initiating atrial tachyarrythmias.^1,2^ Ablation of the earliest activation site and isolation of the SVC are the most described techniques for elimination of the arrhythmogenic focus.^1,2^ However, damage of the right phrenic nerve (PN) is a major complication of this procedure.^3^

## Summary figure

**Figure:**
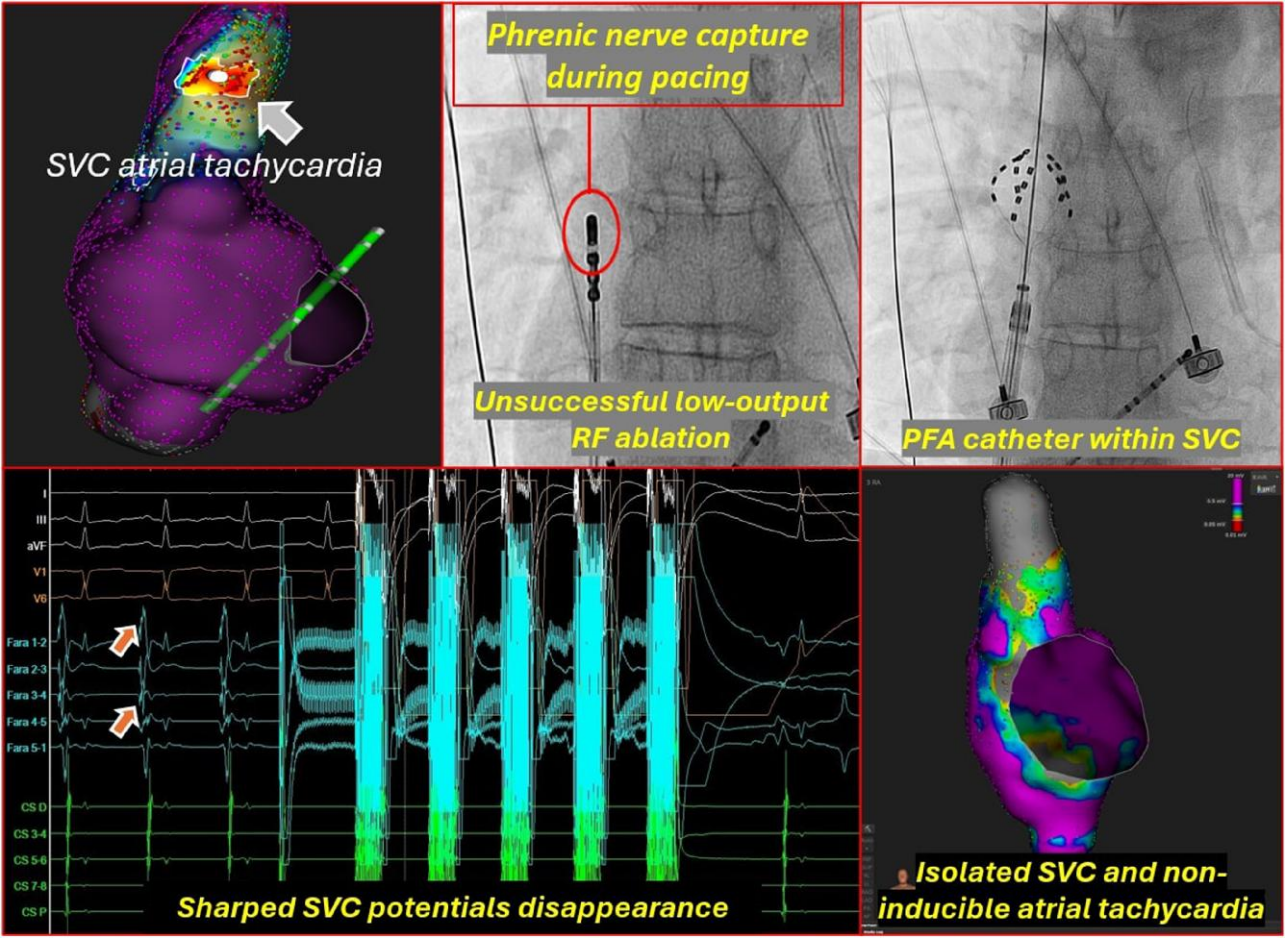


## Case presentation

**Figure 1:**
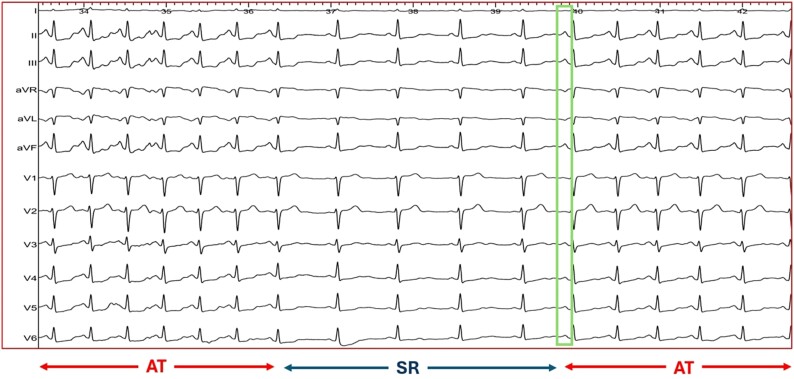
During the procedure, sustained and non-sustained episodes of atrial tachycardia were presented displaying the phenomenon of ‘warm-up’ and ‘cool-down’ during initiation and termination, respectively. *P*-wave was highly positive in leads II, III, aVF, and negative in lead aVL (box).

**Figure 2:**
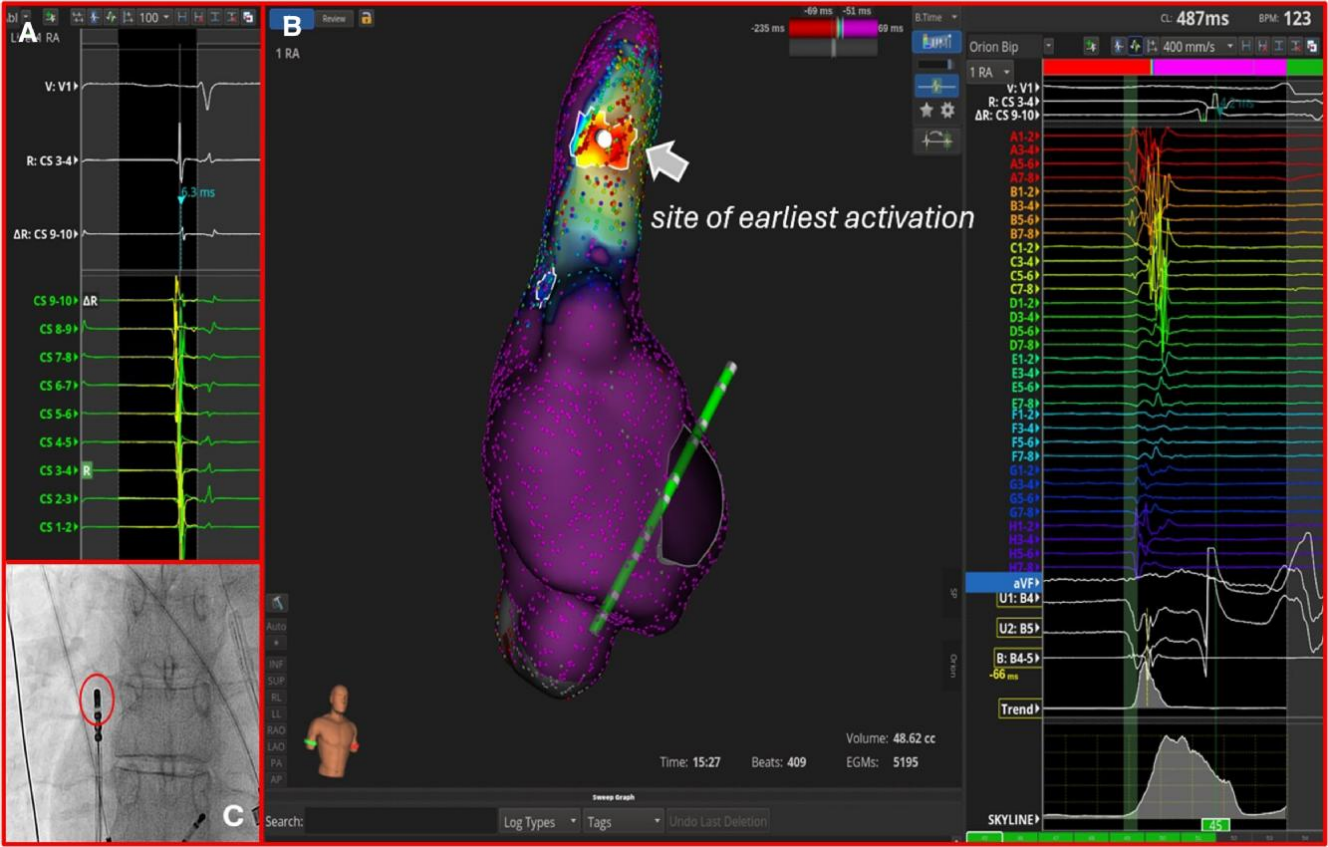
(*A*) Concentric pattern of coronary sinus activation during atrial tachycardia. (*B*) The electroanatomical activation map demonstrated a focal origin located at the lateral aspect of the superior vena cava with radial spread of activation from the earliest activation site. (*C*) High-output pacing (10 V/2.0 ms) from this site resulted in phrenic nerve capture. To avoid phrenic nerve injury, the power output of radiofrequency applications was limited to 20 W.

We report the case of a 41-year-old female patient admitted for catheter ablation of a frequent paroxysmal atrial tachycardia (AT) resistant to antiarrhythmic drugs. *P*-wave was highly positive in leads II, III, aVF and negative in lead aVL (*Figure 1*). She had neither structural heart disease nor known comorbidities predisposing to atrial myopathy. All baseline investigations including laboratory tests, echocardiography vital signs, and physical examination findings were unremarkable. Bisoprolol was interrupted 2 days before the procedure. During the procedure, sustained and non-sustained spontaneous AT episodes (without hemodynamic compromise, blood pressure: 115/75 mmHg) with a cycle length of 485 ms and concentric pattern of activation were presented displaying the phenomenon of ‘warm-up’ and ‘cool-down’ during initiation and termination, respectively (*Figures 1* and *2A*). Endocardial mapping of the right atrium (RA) was performed using the IntellaMap Orion™ mini-basket mapping catheter and the ultra-high density Rhythmia™ mapping system (Boston Scientific, Marlborough, MA, USA). The electroanatomical activation map demonstrated a focal origin located at the lateral SVC aspect with radial spread of activation from the earliest activation site (*Figure 2C*). This site was located ∼17 mm above the anatomical RA-SVC junction where the breakthrough of the sinus node wavefront was defined using electroanatomical mapping during sinus rhythm (*Figure 4C*).

Importantly, high-output pacing (10 V/2.0 ms) from this site resulted in PN capture confirmed by fluoroscopy that showed diaphragmatic movement during pacing. Ablation was performed using a 4.5-mm irrigated-tip ablation catheter (IntellaNav MiFi OI™; Boston Scientific) capable of real-time local impedance monitoring (*Figure 2B*). To avoid PN injury, the power output of radiofrequency (RF) applications was limited to 20 W, and RF delivery was immediately terminated when local impedance values reached the plateau of the impedance curve. Moreover, high-output pacing from the ablation catheter to capture the PN was performed during lesions to observe any weakening of the diaphragmatic contraction. However, low-output RF ablation was insufficient to terminate AT.

**Figure 3:**
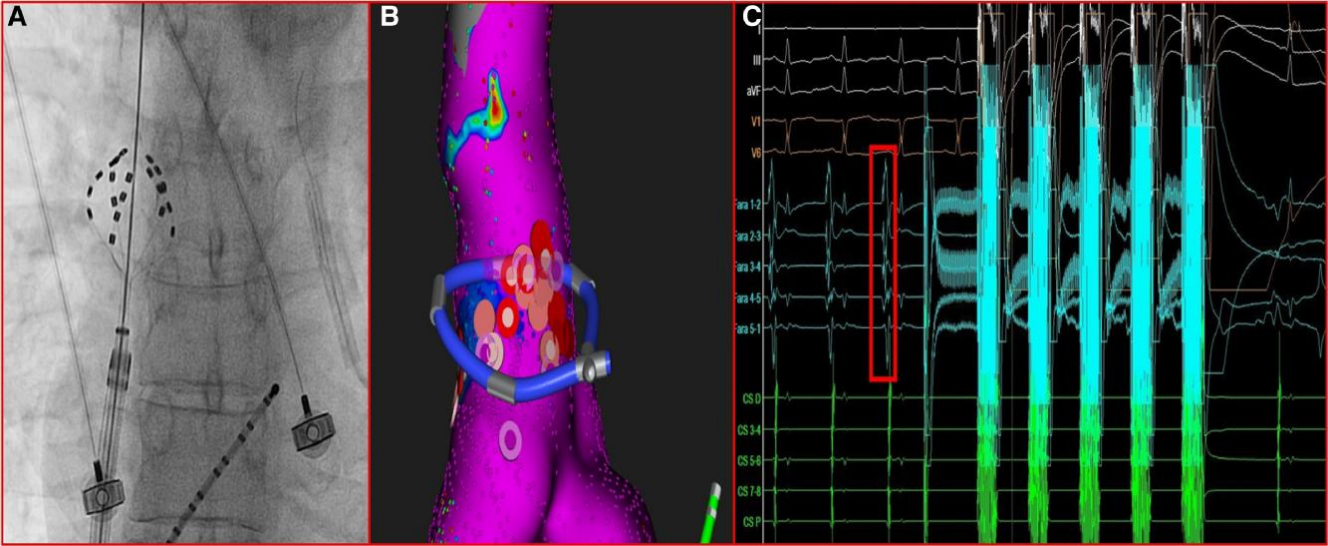
The pentaspline pulsed-field ablation catheter was advanced into the superior vena cava to the level of the earliest activation under fluoroscopic guidance and visualization within the mapping system (*A* and *B*, respectively). (*C*) Two pairs of applications (with a peak voltage of 2.0 kV) were delivered inside the superior vena cava leading to the disappearance of local sharp potentials (box).

To avoid high-power RF applications in proximity to the PN, pulsed-field ablation (PFA) with the FARAPULSE PFA System (Boston Scientific) using the 31-mm pentaspline PFA catheter (Farawave) was decided. The catheter was advanced into the SVC to the level of the earliest activation under fluoroscopic guidance and visualization within the mapping system (*Figure 3*). Two pairs of applications (with a peak voltage of 2.0 kV), in basket configuration, were delivered. After these lesions, AT was not inducible with burst atrial pacing even after intravenous isoproterenol infusion.

**Figure 4:**
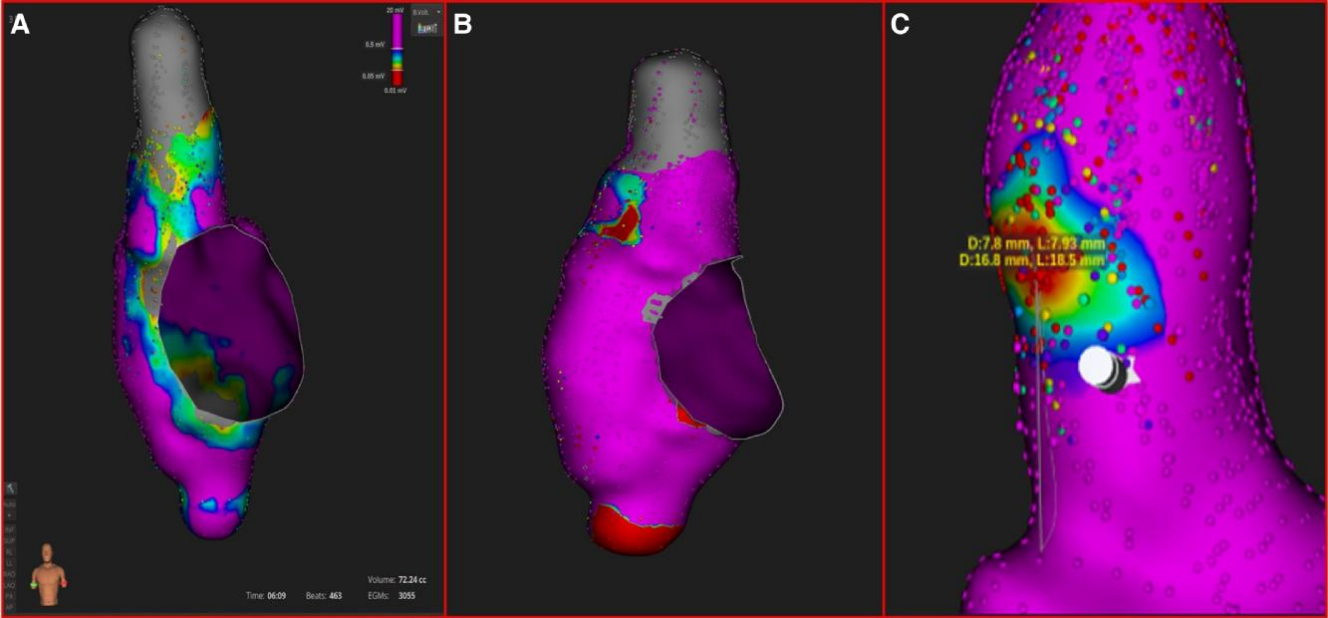
(*A*) Post-ablation voltage mapping revealed superior vena cava isolation. (*B*) Activation mapping revealed the breakthrough of the sinus node during sinus rhythm. (*C*) The focus of the atrial tachycardia was located ∼17 mm above the anatomical right atrium-superior vena cava junction where the breakthrough of the sinus node wavefront was defined.

**Figure 5:**
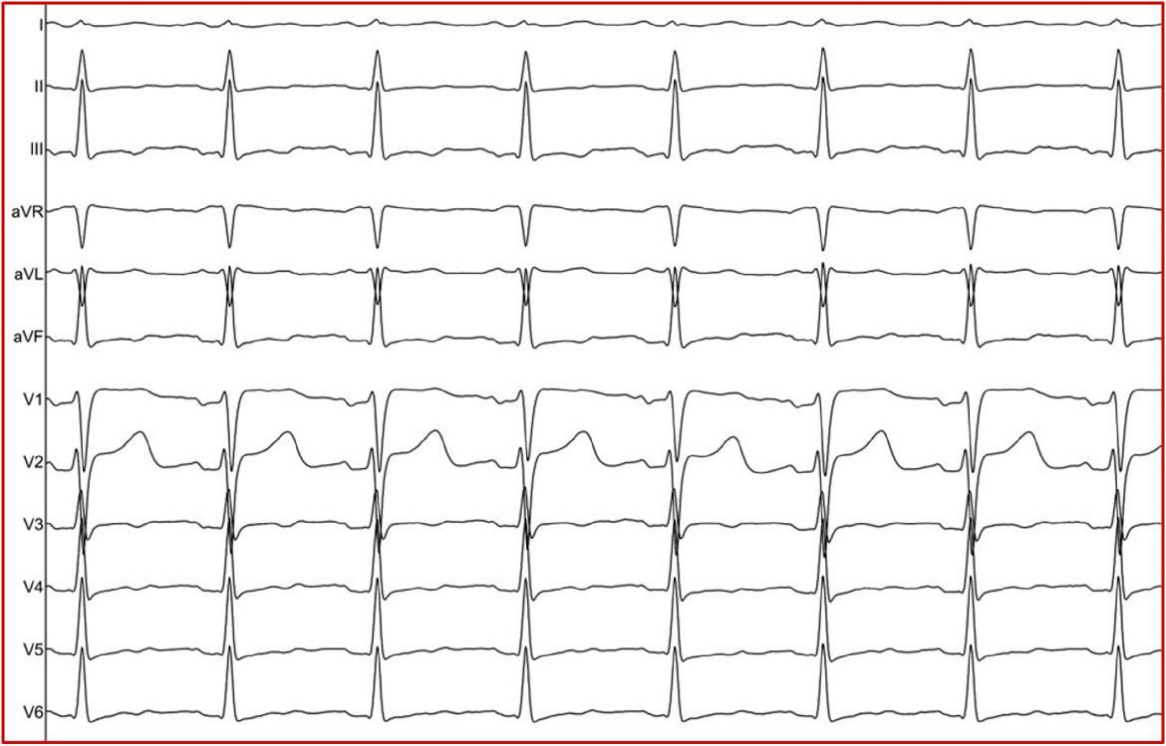
Post-ablation 12-lead electrocardiogram revealed that sinus node function was not compromised.

Post-ablation voltage mapping revealed SVC isolation (*Figure 4A*) while sinus node function was not compromised based on electrocardiogram monitoring (*Figure 5*) and activation mapping (*Figure 4B*). The patient remained free of arrhythmias during 24-h continuous rhythm monitoring and free of symptoms during a 6-month follow-up.

## Discussion

The right PN descends along the right anterolateral SVC surface, and it continues inferiorly to innervate the right diaphragmatic dome.^3^ Due to this proximity to SVC, right PN is vulnerable to injury during ablation procedures within SVC that use thermal ablation modalities.^3,4^

Previous studies have shown that power titration decreases PN injury incidence during RF ablation within SVC and the risk may be rather low when using low-energy delivery at PN capture sites.^3,5^ In this case, low-output ablation, limited to 20 W, was attempted. Moreover, RF delivery was minimized based on the local impedance drop curve. Diaphragmatic movement was not compromised, observed using high-output pacing to capture the PN. However, this approach was insufficient to eliminate AT episodes.

Recent studies have shown that high-power ablation might be effective and safe for SVC isolation when applications are guided by lower ablation index values at lateral SVC segments.^6^ However, further large-scale investigation may be necessary. Moreover, for the irrigated-tip ablation catheter that was used in this case, only low-output ablation has been reported in PN capture sites while the upper power setting is not recommended.^7^

On the other hand, PFA is a novel, non-thermal method for cardiac ablation.^8^ Electrical fields disrupt the cardiomyocyte membrane and its selectivity appears to reduce potential damage to extracardiac tissues such as the adjacent PN. Indeed, initial experience in pulmonary vein isolation with the single-shot pentaspline PFA catheter reported only transient PN injury in addition to an efficacious isolation.^8^

Based on this promising feasibility and safety, several studies have examined the use of this catheter beyond pulmonary vein isolation. In a previous animal study, successful SVC isolation at a dose not predicted to cause PN dysfunction was associated with normal PN function and histopathology.^9^ More recently, SVC isolation with the pentaspline PFA catheter was demonstrated to be feasible and safe in patients undergoing atrial fibrillation ablation.^10^ As not only ablation of the earliest activation site but also SVC isolation may be a successful technique for focal tachycardias originated from the SVC, we performed the latter technique to eliminate the arrhythmogenic focus. To the best of our knowledge, this case is the first report of SVC isolation for a focal AT using this pentaspline PFA catheter. The use of the 35-mm pentaspline PFA catheter for a recurrent reentrant AT originated at the lateral RA has also been reported without complications.^11^ In addition to single-shot devices, focal PFA catheters are also available to increase technique versatility and have recently been examined for atrial arrhythmias ablation.^12^ In a similar case, a focal PFA catheter was successfully used for a RA focal AT in proximity to the PN.^13^

Sinus node may also be damaged during RF SVC isolation due to the proximity of ablation sites to the sinus node.^14^ The definition of the junction of RA and SVC is important, and the ablation of SVC anterolateral free wall should not be close to the SVC orifice.^14^ Transient high-degree sinus node dysfunction also occurred in a recent study using PFA.^10^ In this study, ablation level was based on electrogram recording using the pentaspline catheter. In contrast, electroanatomical mapping with a multi-polar catheter was performed in our case. Importantly, sinus node localization was feasible before ablation as AT was not sustained. In this way, the zone of earliest activation during sinus rhythm was determined while PFA-catheter visualization within the mapping system permitted ablation at a higher level more precisely in comparison to fluoroscopy guidance. Certainly, more data are needed for potential sinus node dysfunction after SVC PFA isolation and to examine the role of electroanatomical mapping to avoid this complication.

Moreover, SVC stenosis can occur after extensive RF ablation.^15^ However, due to its non-thermal nature, there is currently no report of stenosis after PFA-based SVC isolation in similarity with pulmonary vein isolation.^8,10^

## Conclusion

Superior vena cava has been recognized as a source of focal AT. Right PN injury is one of the major complications of thermal ablation within the SVC and low-output RF delivery may be safe but less efficient. This case underscores that non-thermal approaches such as PFA may be preferable to avoid PN injury. However, more data are needed for potential sinus node dysfunction after SVC PFA isolation and for defining the role of electroanatomical mapping while the long-term outcome of PFA in arrhythmias other than atrial fibrillation remains uncertain.

## Data Availability

The data underlying this article cannot be shared publicly due to the privacy of individuals who participated in the study. The data will be shared on reasonable request to the corresponding author.

## Lead author biography

Konstantinos Tampakis is an EHRA-certified cardiac electrophysiology specialist. He graduated from University of Patras Medical School and trained in electrophysiology and device implantation at Clinique Pasteur, Toulouse. He is currently working at the Department of Electrophysiology and Pacing of Henry Dunant Hospital Center in Athens, Greece.

**Consent:** The authors confirm that written consent for the submission and publication of this case report, including images and associated text, was obtained from the patient in accordance with COPE guidance.

**Funding:** This work was supported by the Arrhythmias Institute.
